# The efficacy of high-flow nasal cannula versus non-invasive mechanical ventilation in preventing reintubation in patients at high risk of extubation failure: systematic review and meta-analysis with trial sequential analysis

**DOI:** 10.62675/2965-2774.20260371

**Published:** 2026-05-05

**Authors:** Andrés Esteban Salazar Molina, Héctor Hernández Garcés, Marco Antonio Carangui Urgilés, Omar Patricio Bustamante Celleri

**Affiliations:** 1 Intensive Care Unit Hospital Humanitario San Jose Azogues Cañar Ecuador Intensive Care Unit, Hospital Humanitario San Jose - Azogues, Cañar, Ecuador.; 2 Intensive Care Unit Hospital Doctor Peset Valencia Spain Intensive Care Unit, Hospital Doctor Peset - Valencia, Spain.; 3 Universidad Católica de Cuenca Research Training Department Cuenca Ecuador Research Training Department, Universidad Católica de Cuenca - Cuenca, Ecuador.

**Keywords:** Noninvasive ventilation, Nasal high flow, Airway extubation, Respiratory insufficiency, Hospital mortality, Risk

## Abstract

**Objective:**

To conduct a systematic review and meta-analysis with trial sequential analysis to compare the efficacy of high-flow nasal cannula *versus* noninvasive ventilation in preventing reintubation, post-extubation respiratory failure, mortality, and length of stay in intensive care unit and hospital in patients at high risk of extubation failure.

**Methods:**

A comprehensive literature search was conducted across ten databases: MEDLINE®/PubMed®, Web of Science, SciELO, Embase, Scopus, the Cochrane Central Register of Controlled Trials, the International Clinical Trials Registry Platform, Google Scholar, GreyNet International, and OpenGrey. The primary outcome was reintubation. Secondary outcomes included post-extubation respiratory failure, intensive care unit and hospital mortality, as well as intensive care unit and hospital length of stay.

**Results:**

Ten randomized controlled trials comprising 1,697 patients were included. There were no significant differences between high-flow nasal cannula and non-invasive ventilation in reintubation (RR = 1.00; 95%CI 0.92 - 1.09; p = 0.92; I^2^ = 65.0%), intensive care unit mortality (RR 1.03; 95%CI 1.00 - 1.07; p = 0.08; I^2^ = 28.0%), hospital mortality (RR 1.02; 95%CI 0.99 - 1.06; p = 0.13; I^2^ = 0%), intensive care unit length of stay (mean difference: -0.51 days; 95%CI -1.85 to 0.82; I^2^ = 63.0%), and hospital length of stay (mean difference: 0.80 days; 95%CI -1.72 to 3.32; I^2^ = 47.0%). High-flow nasal cannula showed a non-significant trend toward lower post-extubation respiratory failure and intensive care unit mortality in patients ventilated > 5 days.

**Conclusion:**

In adult patients at high risk of extubation failure, the use of high-flow nasal cannula is not associated with significant differences in reintubation rates, intensive care unit or hospital mortality, or intensive care unit or hospital length of stay compared with noninvasive ventilation.

**PROSPERO register**: CRD420251104362

## INTRODUCTION

The complexity and severity of patients in intensive care units (ICUs) vary widely. It is estimated that 39.0 - 72.0% of ICU patients require invasive mechanical ventilation (IMV).^(1-4)^ Among those extubated, 10.0 - 20.0% require reintubation.^(5,6)^ Extubation failure in critically ill patients increases morbidity, mortality, length of stay (LOS), and healthcare costs.^(6)^ The leading cause is acute respiratory failure.^(7)^

Bocchile et al. reported in a subgroup analysis that high-flow nasal cannula (HFNC) reduced the need for intubation compared with conventional oxygen therapy.^(8)^ Similarly, both HFNC and noninvasive ventilation (NIV) have been shown to lower the incidence of post-extubation respiratory failure and reintubation in patients at low risk of failure.^(9)^

However, evidence in high-risk patients remains inconsistent. Some randomized controlled trials (RCTs) found that HFNC is noninferior to NIV in preventing reintubation,^(10)^ whereas others suggest that NIV is more effective.^(11)^ Two systematic reviews with meta-analyses concluded that NIV reduces reintubation rates, although their populations were heterogeneous, with about 40.0% of patients at high risk.^(12,13)^ Other reviews comparing HFNC and NIV in high-risk patients found no significant differences in reintubation or mortality rates.^(14-16)^ Boscolo et al.^(14)^ and Fernando et al.^(15)^ analyzed this question using subgroup data from only four and six studies, respectively, and excluded patients with IMV > 36 hours, obesity, hypercapnia, or airway disorders. In contrast, Wang et al.^(16)^ directly compared the interventions. However, their sample included many patients with chronic obstructive pulmonary disease (COPD) and did not consider IMV duration as a high-risk criterion.

Unlike NIV, HFNC offers several advantages, including better comfort, lower cost, and fewer adverse effects such as patient self-inflicted lung injury (P-SILI), gastric distension, mask discomfort, claustrophobia, and facial ulcers.^(17,18)^

Given the variability in current protocols and the ongoing difficulty in identifying patients at high risk of extubation failure, we designed this study to evaluate the role and effectiveness of both therapies in this vulnerable group. Our goal was to conduct a systematic review and meta-analysis with trial sequential analysis to compare the efficacy of HFNC *versus* NIV in preventing reintubation, post-extubation respiratory failure, mortality, and LOS in intensive care unit and hospital in patients at high risk of extubation failure.

## METHODS

The Preferred Reporting Items for Systematic Reviews and Meta-Analyses (PRISMA)^(19)^ checklist was used to guide the reporting of the systematic review and meta-analysis, and the study was registered with PROSPERO (CRD420251104362).

### Search strategy

Data management was performed using RevMan software.^(20)^ Two investigators independently searched the MEDLINE®/PubMed®, Web of Science, Embase, Scopus, the Cochrane Central Register of Controlled Trials (CENTRAL), the International Clinical Trials Registry Platform (ICTRP) databases, and gray literature sources, including Google Scholar, GreyNet International, and OpenGrey. The search covered all records in each database from their inception until February 2025. No geographic restrictions were applied. No language restrictions were applied. Corresponding authors were contacted to request unpublished data or clarification of ambiguous information.

### Study selection

The following inclusion criteria were applied: patients at high risk of extubation failure, defined by at least one of the following factors: age over 65 years, cardiac or respiratory disease,^(21-23)^ Acute Physiology and Chronic Health Evaluation II (APACHE II) score ≥ 12 at the time of extubation,^(21,22)^ body mass index (BMI) ≥ 30kg/m^2^,^(24)^ upper airway obstruction, weak cough, two or more comorbidities, more than one failed spontaneous breathing trial, arterial carbon dioxide pressure (PaCO_2_) ≥ 45mmHg after extubation,^(21)^ and IMV ≥ 7 days.^(24)^ If studies included mixed populations (high- and low-risk), we extracted subgroup information from each study, if the information was not available in the main text, supplementary material, or after contacting the authors, we excluded the study; use of HFNC and NIV as interventions; reintubation reported as a primary or secondary outcome; and RCTs as the study design.

The exclusion criteria included: pregnancy; patients receiving extracorporeal membrane oxygenation (ECMO); treatment with hyperbaric oxygen; and non-RCT studies.

### Data extraction and quality assessment

Details about Boolean operators with Medical Subject Headings (MeSH) terms are summarized in table 1S (Supplementary Material). Data was independently extracted by two researchers, and any discrepancies were resolved by a third reviewer. From the selected studies, we collected the following information: first author, year of publication, country, sample size, type of study (single-center or multicenter), cause of IMV, duration of IMV, definition of high-risk extubation failure, intervention and control measures, post-extubation respiratory failure, reintubation, ICU mortality, hospital mortality, LOS in the ICU and hospital.

The risk of bias was assessed using the Cochrane Risk of Bias 2 tool (RoB 2).^(25,26).^ Studies were considered high risk if at least one domain was rated high risk; otherwise, they were classified as low risk. Two researchers independently assessed the certainty of the pooled results using the GRADE (Grading of Recommendations Assessment, Development and Evaluation) approach. The meta-analysis was evaluated across five domains: risk of bias, inconsistency, indirectness, imprecision, and publication bias. The certainty of evidence was rated as high, moderate, low, or very low.^(27)^

### Outcome measures

The primary endpoint was the overall reintubation rate, defined as any reintubation event within 7 days after extubation. This outcome was used to compare the efficacy of HFNC and NIV in critically ill patients at high risk of extubation failure.

Secondary outcomes included ICU and hospital mortality, ICU and hospital LOS, and post-extubation respiratory failure. The latter was defined as the presence or persistence of at least one of the following: respiratory acidosis (pH < 7.35 with PaCO_2_ ≥ 45mmHg), peripheral oxygen saturation (SpO_2_) < 90% or partial pressure of oxygen in arterial blood (PaO_2_) < 60mmHg with fraction of inspired oxygen (FiO_2_) > 0.4, respiratory rate ≥ 35/minute, decreased level of consciousness, agitation, inability to clear secretions, or clinical signs of respiratory muscle fatigue or increased work of breathing (use of accessory muscles, paradoxical abdominal motion, or intercostal retraction).

### Statistical analysis

Data were analyzed using Statistical Package for the Social Sciences (SPSS) version 30 for descriptive statistics, and Review Manager (RevMan, Cochrane Collaboration) was used to perform both fixed- and random-effects meta-analyses, with forest plots generated to visualize the pooled estimates. Dichotomous outcomes were expressed as risk ratios (RR) with 95% confidence intervals (95%CI), while continuous outcomes were reported as mean differences with 95%CI. A p value < 0.05 was considered statistically significant. To assess heterogeneity among studies, Cochran's Q test was applied, with heterogeneity indicated by a p value < 0.1. The I^2^ statistic was used with cutoffs of 25% (low), 50% (moderate), 75% (high), and >75% (very high). An L´Abbé plot was also used to visually assess heterogeneity in dichotomous outcomes. Publication bias was assessed using funnel plots and Egger's test (when at least ten studies were available).

Subgroup analyses were performed based on duration of IMV (≤ 2 days, 3 - 5 days, 5 - 7 days, > 7 days), duration of each treatment (≤ 24 hours, ≤ 48 hours) and cause of intubation (hypercapnic or hypoxemic respiratory failure); sensitivity analyses were conducted by excluding studies with high risk of bias and meta-regression based on the number of high-risk factors for extubation failure (1, 2, 3, or ≥ 4).

Finally, to balance type I and type II errors and to evaluate whether the observed effects could change with future studies, we performed a trial sequential analysis (TSA) using version 0.9 Beta of the Copenhagen Trial Unit software.

For binary outcomes, we applied a random-effects model (DerSimonian-Laird) to construct the Z-curve, assuming a type I error of 5%, a relative risk reduction of 20%, and a statistical power of 80%. For continuous outcomes, the Z-curve was built with a type I error of 5%, a power of 80%, a variance of 20, and mean differences of 2 and 5 days for ICU and hospital LOS, respectively. These parameters were used to calculate the required information size to confirm or refute the intervention effect.

The variance of 20 was derived from the average standard deviation across trials, while the mean difference reflected a clinically relevant effect reported in previous studies. In both analyses, we used the O’Brien-Fleming method to adjust significance thresholds. TSA was applied to both primary and secondary outcomes.^(28,29)^

## RESULTS

### Study selection and characteristics

**Figure 1:**
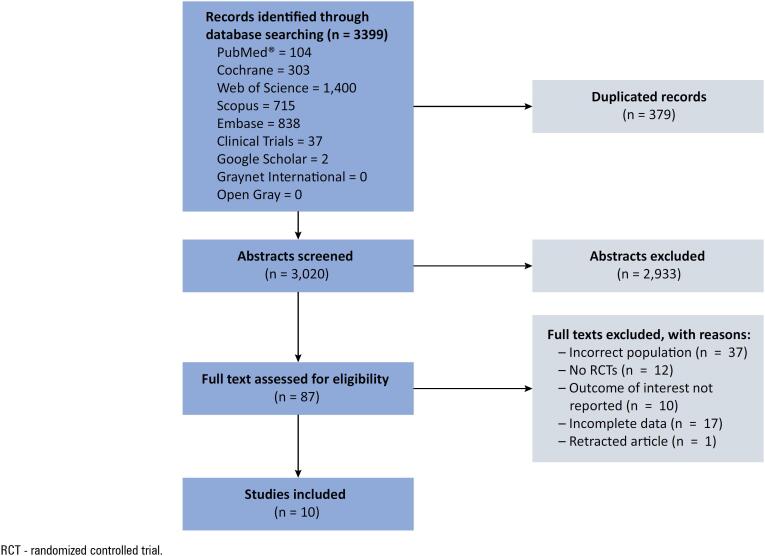
Diagram of selected studies.

A total of 3,399 studies were identified through the search strategy. After removing 379 duplicates, 3,020 records were screened by title and abstract, and 87 were selected for full-text review. Finally, 10 RCTs were included^(30-39)^ (Figure 1). The RCTs were published between 2015 and 2024, including a total of 1,697 patients, of whom 844 (49.7%) received HFNC and 853 (50.3%) received NIV. Details of the included RCTs are summarized in table 1.

**Table 1:** Basic characteristics of the included studies

Study	Design	Country (year)	Population (n/male/age)		High risk criteria	Intervention duration (hours)	Outcomes
			HFNC	NIV			
Tseng et al.^(30)^	Single center Mixed population	Taiwan (2023)	20/13 73 ± 13	20/10 74 ± 11	APACHE II ≥ 12 at extubation; heart failure as primary indication for IMV; age ≥ 65 years; COPD; BMI ≥ 30kg/m^2^; airway patency problems; inability to clear respiratory secretions; difficult or prolonged weaning	≤ 24	1, 3, 4, 5, 6
Temphattharachok et al.^(31)^	Single center Mixed population	Thailand (2024)	17/NA NA	17/NA NA	≥ 65 years; chronic cardiac or pulmonary disease	NA	1
Kumari et al.^(32)^	Single center Mixed population	India (2024)	30/21 53 ± 16	30/21 53 ±18	Chronic cardiac and/or pulmonary disease; ≥ 2 comorbidities	24 - 48	1
Hernández et al.^(33)^	Multicenter Mixed population	Spain (2016)	314/202 64 ± 15	290/186 64 ± 15	APACHE II ≥ 12 at extubation; heart failure; ≥ 65 years; COPD; BMI ≥ 30kg/m^2^; inability to clear respiratory secretions; laryngeal edema; ≥ 1 comorbidity, IMV ≥ 7 days	≤ 24	1, 2, 3, 4, 5, 6
Magdy et al.^(34)^	Single center COPD patients	Egypt (2021)	120/76 68.4 ± 6.8	110/64 67.9 ± 6.9	APACHE II ≥ 12 at extubation; heart failure; ≥ 65 years; COPD; BMI ≥ 30kg/m^2^; inability to clear respiratory secretions; IMV ≥ 7 days	NA	1, 2, 3, 4, 5, 6
Hernández et al.^(35)^	Multicenter Mixed population	Spain (2022)	92/67 60.9 ± 14.3	90/50 59.9 ± 15.4	APACHE II ≥ 12 at extubation; heart failure; ≥ 65 years; COPD; BMI ≥ 30kg/m^2^; inability to clear respiratory secretions; airway patency problems; ≥ 2 comorbidities, IMV ≥ 7 days; difficult or prolonged weaning; hypercapnia at the end of the SBT	24 - 48	1, 2, 3, 4, 5, 6
Theerawit et al.^(36)^	Single center Mixed population	Thailand (2021)	71/35 68.2 ± 18.7	69/28 71.2 ± 16.0	Heart failure; ineffective cough; COPD; SBT failure; APACHE II ≥ 12 at extubation; airway patency problems	≤ 24	1, 2, 4
Hernández et al.^(37)^	Single center Mixed population	Spain (2024)	72/33 NA	72/32 NA	≥ 65 years; heart failure; COPD; IMV ≥ 7 days; BMI ≥ 30kg/m^2^	24 - 48	1, 2, 3, 4, 5, 6
Tongyoo et al.^(38)^	Multicenter Septic patients	Thailand (2021)	110/60 60 ± 17.5	112/61 62.6 ± 18.03	APACHE II ≥ 12 at extubation	≤ 24	1, 3, 4, 5, 6
Jing et al.^(39)^	Single center Chronic respiratory disease	China (2019)	22/NA 77.4 ± 6.8	20/NA 73.9 ± 6.9	PaCO_2_ ≥ 45mmHg at extubation	24 - 48	1, 2, 4, 5

HFNC - high-flow nasal cannula; NIV - noninvasive ventilation; APACHE -Acute Physiology and Chronic Health Evaluation Classification System; IMV - invasive mechanical ventilation; COPD - chronic obstructive pulmonary disease; BMI - body mass index; NA - no available; SBT - spontaneous breathing trial; PaCO_2_ - arterial carbon dioxide pressure. Outcomes: 1 - reintubation; 2 - post-extubation respiratory failure; 3 - intensive care unit mortality; 4 - hospital mortality; 5 - length of stay in intensive care unit; 6 - length of stay in hospital.

Except for the study by Temphattharachok et al.,^(31)^ which was classified as high risk of bias, all other RCTs were categorized as low risk of bias (Figure 1S - Supplementary Material). Certainty of evidence (GRADE) was high for postextubation respiratory failure at 7 days, ICU mortality at 7 days, and 28-day mortality; moderate for reintubation at 7 days; and low for ICU and hospital LOS (Table 2).

**Table 2:** Assessment of the certainty of evidence for each outcome using the GRADE methodology, considering risk of bias, inconsistency, indirectness, imprecision, and publication bias

Outcome	# Studies	Certainty of the evidence					# Patients		Effect		Certainty
		Risk of bias	Inconsistency	Indirectness	Imprecision	Other considerations	HFNC	NIV	Relative effect (95%CI)	Absolute effect (95%CI)	
Reintubation (7 days; measured by # patients requiring reintubation)	10 RCTs (Tseng et al. ^(30)^; Temphattharachok et al.^(31)^; Kumari et al.^(32)^; Hernández et al.^(33)^; Magdy et al.^(34)^; Hernández et al.^(35)^; Theerawit et al.^(36)^; Hernández et al.^(37)^; Tongyoo et al.^(38)^; Jing et al.^(39)^)	No serious concern	No serious concern	No serious concern	No serious concern	Strong suspicion of publication bias	171/844 (20.3)	179/853 (21.0)	RR 1.00 (0.94 to 1.06)	0 fewer per 1,000 (from 13 fewer to 13 more)	⨁⨁⨁◯ Moderate
Post-extubation respiratory failure (7 days; adverse events)	6 RCTs (Tseng et al.^(30)^; Hernández et al.^(33)^; Magdy et al.^(34)^; Hernández et al.^(35)^; Tongyoo et al.^(38)^; Jing et al.^(39)^)	No serious concern	No serious concern	No serious concern	No serious concern	None	185/844 (21.9)	240/853 (28.1)	RR 1.06 (1.01 to 1.11)	17 more per 1,000 (from 3 more to 31 more)	⨁⨁⨁⨁ High
ICU mortality (7 days; ICU deaths)	6 RCTs (Tseng et al. ^(30)^; Hernández et al.^(33)^; Magdy et al.^(34)^; Hernández et al.^(35)^; Tongyoo et al.^(38)^); Jing et al.^(39)^)	No serious concern	No serious concern	No serious concern	No serious concern	None	42/844 (5.0%)	63/853 (7.4)	RR 1.03 (1.00 to 1.07)	2 more per 1000 (from 0 to 5 more)	⨁⨁⨁⨁ High
Hospital mortality (28 days)	8 RCTs (Tseng et al.^(30)^; Temphattharachok et al.^(31)^; Kumari et al.^(32)^; Hernández et al.^(33)^; Magdy et al.^(34)^; Hernández et al.^(35)^; Tongyoo et al.^(38)^; Jing et al.^(39)^)	No serious concern	No serious concern	No serious concern	No serious concern	None	100/844 (11.8)	119/853 (14.0)	RR 1.02 (0.99 to 1.06)	3 more per 1,000 (from 1 fewer to 8 more)	⨁⨁⨁⨁ High
ICU length of stay (7 days)	7 RCTs (Tseng et al.^(30)^; Temphattharachok et al.^(31)^; Hernández et al.^(33)^; Magdy et al.^(34)^; Hernández et al.^(35)^; Tongyoo et al. ^(38)^; Jing et al.^(39)^)	No serious concern	Serious	Serious	No serious concern	None	844	853	MD 0.51 days shorter (from 1.85 shorter to 0.82 longer)	—	⨁⨁◯◯ Low
Hospital length of stay (28 days)	6 RCTs (Tseng et al.^(30)^; Hernández et al.^(33)^; Magdy et al.^(34)^; Hernández et al.^(35)^; Tongyoo et al.^(38)^; Jing et al.^(39)^)	No serious concern	No serious concern	Serious	Serious	None	844	853	MD 0.8 days longer (from 1.72 shorter to 3.32 longer)	—	⨁⨁◯◯ Low

HFNC - high-flow nasal cannula; NIV - noninvasive ventilation; 95%CI - 95% confidence interval; RCT - randomized clinical trial; RR - risk ratio; ICU - intensive care unit; MD - mean difference.

### Reintubation

**Figure 2:**
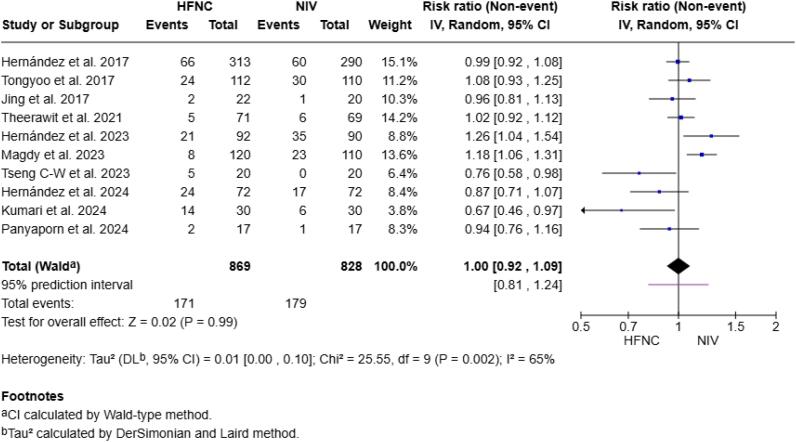
Forest plot comparing the effect of high-flow nasal cannula *versus* noninvasive ventilation on the risk of reintubation.

Efficacy regarding reintubation rates was reported in all ten studies. In the HFNC group, 171 patients (20.3%) were reintubated, compared with 179 patients (21.0%) in the NIV group. No statistically significant differences were found between the groups (RR 1.00; 95%CI 0.92 - 1.09; p = 0.99; I^2^ = 65.0%; Figure 2). In the influence plot, Magdy et al.^(34)^ study emerged as a potential outlier, with notably harmful studentized residuals and an effect that increases the variance of the pooled estimate (Figure 2SB - Supplementary Material).

The funnel plot showed asymmetry in favor of HFNC (Figure 2SA and 3S - Supplementary Material), and Egger's test was not significant (p = 0.105).

**Figure 3:**
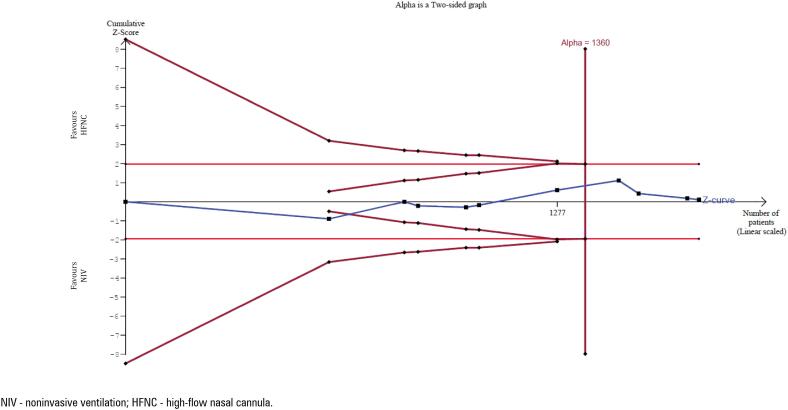
Trial sequential analysis of reintubation.

In TSA, the Z-curve reached the required sample size (RIS) (1,360 patients) and crossed the futility boundary, suggesting that HFNC, compared with NIV, does not reduce the relative risk by 20.0% or more (Figure 3).

The cumulative Z-curve (blue line) crossed the required information size (vertical red line at 1,360 patients) without reaching the trial sequential monitoring boundaries (red lines). A random-effects model was applied, assuming a type I error of 5%, 80% statistical power, and a 20% relative risk reduction. The O’Brien–Fleming α-spending function was used to adjust the significance boundaries and control for type I error due to repeated significance testing. These results indicate that the required information size has been achieved, and further trials are unlikely to change the conclusion that a 20% relative risk reduction in reintubation is not present.

### Post-extubation respiratory failure

**Figure 4:**
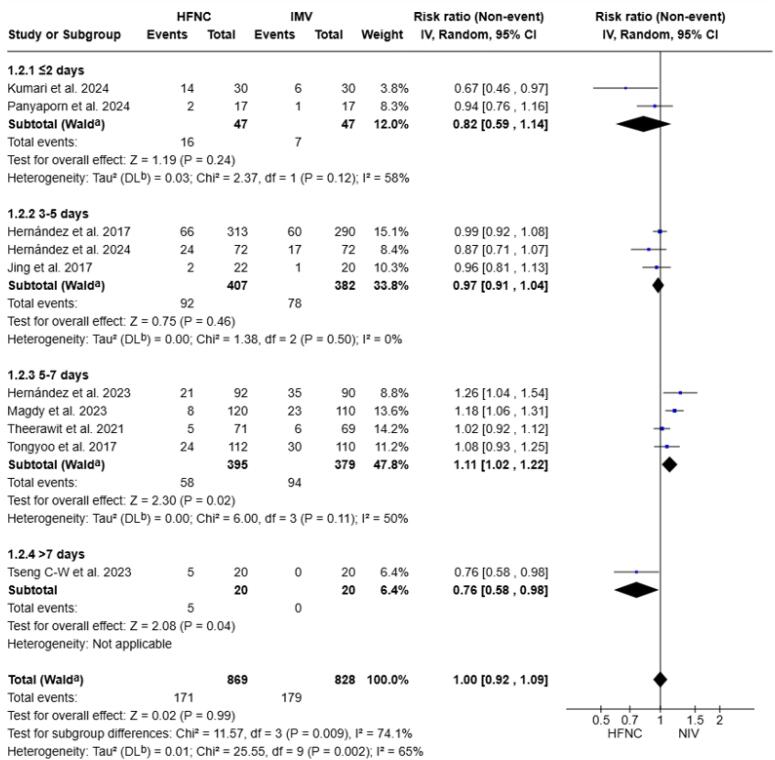
Forest plot comparing the effect of high-flow nasal cannula *versus* noninvasive ventilation on reintubation risk stratified by the duration of invasive mechanical ventilation.

**Figure 5:**
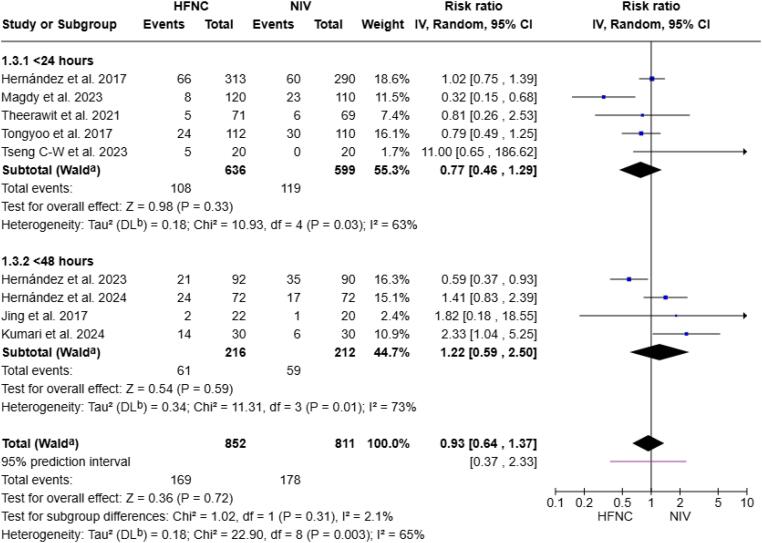
Forest plot comparing the effect of high-flow nasal cannula *versus* noninvasive ventilation on reintubation risk, stratified by intervention duration.

Six studies reported this outcome.^(30-34,36)^ Post-extubation respiratory failure was more frequent in the NIV group (RR 1.06; 95%CI 1.01 - 1.11; p = 0.04; I^2^ = 27.0%; Figure 4S [Supplementary Material]). The funnel plot showed asymmetry, suggesting potential small-study effects in both directions (Figure 5S - Supplementary Material).

In TSA, the Z-curve reached the RIS and crossed the boundary in favor of HFNC, indicating a relative risk reduction of 20.0% (Figure 6S - Supplementary Material).

### Mortality

Six studies reported ICU mortality,^(27,30,32-34)^ with no significant differences between groups (RR 1.03; 95%CI 1.00 - 1.07; p = 0.08; I^2^ = 28.0%; Figure 7SA [Supplementary Material]). In TSA, the Z curve did not reach the alpha limit (Figure 7SB - Supplementary Material). Twenty-eight-day mortality was reported in eight studies,^(27,30-33,36)^ and the meta-analysis showed no statistically significant differences (RR 1.02; 95%CI 0.99 - 1.06; p = 0.13; I^2^ = 0%; Figure 8SA [Supplementary Material]). Only one study reported 90-day mortality. The Z curve in TSA reached the RIS and did not cross the boundary line (Figure 8SB - Supplementary Material).

### Length of stay

Seven studies reported ICU LOS,^(27,30-32,34-36)^ with no significant differences between groups (mean difference: -0.51 days; 95%CI -1.85 to 0.82; p = 0.45; I^2^ = 63.0%; Figure 9SA [Supplementary Material]). Z curve reached RIS (Figure 9SB - Supplementary Material).

Hospital LOS was reported in six studies,^(27,30-32,34,35)^ and no significant differences were found (mean difference: 0.80 days; 95%CI: -1.72 to 3.32; p = 0.53; I^2^ = 47.0%; Figure 10SA [Supplementary Material]), and in TSA, the Z curve reached the RIS Figure 10SB (Supplementary Material).

### Subgroup analysis and meta-regression

Subgroup analyses of primary and secondary outcomes were performed according to IMV duration (< 2 days, 3 - 5 days, 5 - 7 days, > 7 days), treatment duration (< 24 hours, 24 - 48 hours, > 48 hours), and cause of intubation. We also performed a meta-regression based on the number of high-risk factors for extubation failure (1 - 2, 3, or ≥ 4).^(39,40)^

Our results did not show significant differences in reintubation rates stratified by the duration of IMV, intervention duration, or cause of intubation (Figures 4 and 5, Figure 11S [Supplementary Material]), nor by the number of risk factors (Table 2S [Supplementary Material]).

For post-extubation respiratory failure, subgroup analyses according to duration of treatment and cause of intubation showed no significant differences (Figures 12S and 13S - Supplementary Material). In contrast, subgroup analysis by duration of IMV (3 – 5 days) showed a higher incidence in the NIV group (RR 1.08; 95%CI 1.01–1.16; p = 0.03; I2 = 32%; Figure 14S [Supplementary Material]).

In the subgroup of patients with IMV duration between 5 - 7 days, ICU mortality was higher in the NIV group (RR 1.06; 95%CI 1.02 - 1.10; p = 0.01; I^2^ = 28.0%, Figure 4). No significant differences were observed in the other subgroups. Similarly, for hospital mortality, ICU LOS, and hospital LOS, no significant differences were observed across subgroups (Tables 3S and 4S - Supplementary Material).

### Sensitivity analysis

A sensitivity analysis was performed, excluding the study by Temphattharachok et al.^(31)^ because of its high risk of bias. This analysis evaluated reintubation, post-extubation respiratory failure, ICU and hospital mortality, and ICU and hospital LOS. No significant changes were observed in heterogeneity or pooled results, except for post-extubation respiratory failure, which became statistically non-significant (RR 1.04; 95%CI 0.99 - 1.09; p = 0.12; I^2^ = 22.0%; Table 4S [Supplementary Material]).

For the reintubation outcome, the funnel plot showed that the study by Magdy et al.^(34)^ displayed marked asymmetry and fell outside the confidence interval, suggesting a possible overestimation of the effect. Excluding this study reduced heterogeneity from I^2^ = 65% to 0%, identifying it as the primary source of variability across trials.

## DISCUSSION

In this systematic review and meta-analysis of patients at high risk of extubation failure, HFNC was associated with a similar risk of reintubation as NIV. Although substantial heterogeneity was observed (with Magdy et al.^(34)^ identified as a potential outlier in the influence plot), this is likely due to differences in patient characteristics (e.g., causes of intubation, duration of mechanical ventilation, and concomitant treatments) and variability in sample sizes across studies.

Intensive care unit and hospital mortality, as well as ICU and hospital LOS, were similar between HFNC and NIV. High-flow nasal cannula also showed a non-significant trend toward lower post-extubation respiratory failure and ICU mortality in patients ventilated for more than 5 days.

In the TSA, using a predefined relative risk reduction of 20% for dichotomous outcomes, the RIS was reached for reintubation, post-extubation respiratory failure, and hospital mortality, but not for ICU mortality. For continuous outcomes, with a mean difference of 2 days (ICU LOS) and 5 days (hospital LOS), the RIS was achieved.

Our results are consistent with those of other meta-analyses. For example, the review by Guo et al., which included 1,746 patients from six RCTs, found no significant differences in reintubation rates between the two groups. However, HFNC was associated with a reduced incidence of post-extubation respiratory failure.^(41)^ Notably, patients in that review were not stratified by risk of reintubation, and the primary focus was on extubated patients with respiratory insufficiency. In the review by Al Nufaiei et al., no differences were found between the two treatments. However, the study was conducted exclusively in patients with COPD, and a meta-analysis was not performed.^(42)^ In the study by Feng et al., among patients with acute exacerbation of COPD, NIV was superior to HFNC in preventing reintubation in non-hypercapnic patients (baseline arterial carbon dioxide pressure < 50mmHg). Although the study included only 612 patients, not all were considered at high risk of extubation failure.^(43)^ In our review, we observed differences compared to the previously cited studies, as we included patients who had been receiving IMV for at least 24 hours and presented various high-risk factors for extubation failure. Our population was heterogeneous, with different causes of intubation, which may have contributed to greater disease severity and a higher reintubation rate in both groups.

In our study, HFNC showed outcomes similar to NIV for reintubation rate, hospital mortality, and ICU and hospital LOS. It may also offer potential benefits in reducing post-extubation respiratory failure and ICU mortality in patients who received IMV for more than 5 days. These findings suggest that HFNC can be considered as an alternative when NIV is unavailable or contraindicated, particularly in cases of prolonged IMV.

Finally, the use of TSA adds further robustness to our findings. For key outcomes –

reintubation, post-extubation respiratory failure, and hospital mortality – the RIS was achieved, indicating that the current evidence is sufficiently powered to detect a clinically meaningful 20% relative risk reduction. This suggests that additional randomized trials on these endpoints may be unnecessary, reflecting a mature evidence base and potential futility of further research.

In contrast, TSA for ICU mortality did not reach the RIS, highlighting the need for more high-quality studies to clarify this outcome. Overall, our results and TSA findings support the reliability of HFNC as a valid alternative to NIV in high-risk patients after extubation. This evidence may help guide future clinical practice guidelines and support the integration of HFNC into standardized post-extubation management protocols.

Among the strengths of our study, we highlight extensive bibliographic research, inclusion of a heterogeneous patient population, and a low overall risk of bias. Furthermore, our methodology was robust: we applied TSA to control types I and II errors, conducted subgroup analyses, meta-regression, and sensitivity analyses, and evaluated the certainty of evidence for each outcome using the GRADE approach. These strengths provide a solid framework that increases the reliability of our findings. Significantly, by demonstrating that HFNC offers comparable outcomes to NIV and potential benefits in specific subgroups, our study contributes valuable evidence to an area where clinical uncertainty persists. This reinforces the clinical relevance of HFNC in post-extubation management and may help guide both current practice and future guideline recommendations.

However, our study has some limitations. The main limitation concerns the selection of the study population, as many of the included trials were not conducted exclusively in high-risk patients. For example, in the study by Tongyoo et al.,^(38)^ the main population consisted of patients with sepsis or septic shock, and high-risk patients were selected based on criteria such as APACHE II score ≥ 12 on the day of extubation, BMI ≥ 30kg/m^2^, ≥ 65 years, and duration of intubation > 7 days. Second, the duration of interventions varied across studies, and the criteria used to define high-risk patients were inconsistent. However, we addressed this heterogeneity through subgroup analyses and meta-regression. Third, the studies employed different criteria for defining failure of HFNC or NIV treatment and the need for reintubation. Fourth, given that obesity is a well-established risk factor for extubation failure and most of the included trials were conducted in Asia, where obesity prevalence is low, this factor may limit the generalizability of the findings. In our cohort, obesity prevalence was 15.1% in the HFNC group and 15.9% in the NIV group, which may be particularly relevant in Western populations, where obesity rates are substantially higher.

## CONCLUSION

In adult patients at high risk of extubation failure, high-flow nasal cannula showed no significant differences compared with noninvasive ventilation in reintubation rates, intensive care unit or hospital mortality, or intensive care unit and hospital length of stay. High-flow nasal cannula may also reduce the incidence of post-extubation respiratory failure; however, this potential benefit was not consistently confirmed in subgroup analyses.

## Data Availability

The datasets analyzed during the current study were obtained from previously published studies cited in the reference list. The data extraction forms and statistical analysis files are available from the corresponding author upon reasonable request.
