# Supplementary material for: The efficacy of high-flow nasal cannula versus non-invasive mechanical ventilation in preventing reintubation in patients at high risk of extubation failure: systematic review and meta-analysis with trial sequential analysis
Source: Crit Care Sci. 2026 May 5;38:e20260371. doi: 10.62675/2965-2774.20260371 (PMC13155773; doi:10.62675/2965-2774.20260371)
Supplement: Supplementary Material [file 2965-2774-ccsci-38-e20260371-Mat-suppl.pdf]

Critical Care Science
https://doi.org/10.62675/2965-2774.20260371

SUPPLEMENTARY MATERIAL

The efficacy of high-flow nasal cannula versus non-invasive mechanical ventilation in preventing reintubation in patients at high risk of extubation failure: systematic review and meta-analysis with trial sequential analysis

Andrés Esteban Salazar Molina[1], Héctor Hernández Garcés[2], Marco Antonio Carangui Urgilés[1], Omar Patricio Bustamante Celleri[3]

Table 1S - Search strategies by database

Database	Search strategy
PubMed	((((((“extubation”[All Fields]) AND (“extubation failure”[All Fields])) OR (“respiratory failure postextubation”[All Fields])) OR (“high risk patient”[All Fields])) AND (“noninvasive mechanical ventilation”[All Fields] OR “noninvasive mechanical ventilation nmv”[All Fields] OR “noninvasive mechanical ventilation therapy”[All Fields] OR “noninvasive mechanical ventilation treatment”[All Fields])) OR (“high flow nasal oxygen”[All Fields] OR “high flow nasal oxygen cannula”[All Fields] OR “high flow nasal oxygen delivery”[All Fields] OR “high flow nasal oxygen group”[All Fields] OR “high flow nasal oxygen groups”[All Fields] OR “high flow nasal oxygen therapy”[All Fields] OR “high flow nasal oxygenation cannula”[All Fields] OR “high flow nasal oxygenation group”[All Fields] OR “high flow nasal oxygenation systems”[All Fields] OR “high flow nasal oxygenation therapy”[All Fields]))) AND (randomized controlled trial[Publication Type])
Web of Science	TS=(“extubation” OR “extubation failure” OR “respiratory failure postextubation” OR “high risk patient”) AND TS=(“noninvasive mechanical ventilation” OR “non invasive ventilation” OR “non invasive mechanical ventilation” OR “noninvasive ventilation” OR “NIV” OR “high flow nasal oxygen” OR “high flow nasal cannula” OR “HFNO” OR “high flow nasal oxygen delivery”) NOT TS=(“systematic review” OR “meta-analysis”)
EMBASE	‘high risk patient’:ti OR ‘extubation’:ti OR ‘extubation failure’:ti) AND (‘noninvasive ventilation’:ti OR ‘noninva sive ventilation’:ti OR ‘high flow nasal cannula therapy’:ti) AND (‘clinical article’/de OR ‘clinical study’/de OR ‘clinical trial’/de OR ‘comparative effectiveness’/de OR ‘comparative study’/de OR ‘controlled clinical trial’/de OR ‘controlled study’/de OR ‘human’/de OR ‘major clinical study’/de OR ‘multicenter study’/de OR ‘multicenter study topic’/de OR ‘randomized controlled trial’/de OR ‘randomized controlled trial topic’/de) AND ([adult]/lim OR [aged]/lim OR [middle aged]/lim OR [very elderly]/lim OR [young adult]/lim)
Scopus	extubation AND failure OR respiratory AND failure AND postextubation OR high AND risk OR reintubation AND noninvasive AND mechanical AND ventilation OR nimv OR non AND invasive AND mechanical AND ventilation OR high AND flow AND nasal AND cannula OR high AND flow AND oxygen OR high AND flow AND nasal AND (LIMIT-TO (DOCTYPE , “ar”) OR LIMIT-TO (DOCTYPE , “re”) OR LIMIT-TO (DOCTYPE , “cp”) OR LIMIT-TO (DOCTYPE , “ed”) OR LIMIT-TO (DOCTYPE , “sh”) OR LIMIT-TO (DOCTYPE , “no”) OR LIMIT-TO (DOCTYPE , “le”)) AND (LIMIT-TO (SUBJAREA , “MEDI”) OR LIMIT-TO (SUBJAREA , “NURS”) OR LIMIT-TO (SUBJAREA , “HEAL”)) AND (EXCLUDE (EXACTSRCTITLE , “Cochrane Database Of Systematic Reviews”) OR EXCLUDE (EXACTSRCTITLE , “Frontiers In Pediatrics”) OR EXCLUDE (EXACTSRCTITLE , “Seminars In Fetal And Neonatal Medicine”) OR EXCLUDE (EXACTSRCTITLE , “Neonatology”) OR EXCLUDE (EXACTSRCTITLE , “Cochrane Database of Systematic Reviews”) OR EXCLUDE (EXACTSRCTITLE , “Frontiers in Pediatrics”) OR EXCLUDE (EXACTSRCTITLE , “Pediatric Critical Care Medicine”) OR EXCLUDE (EXACTSRCTITLE , “Clinics in Perinatology”) OR EXCLUDE (EXACTSRCTITLE , “Seminars in Fetal and Neonatal Medicine”) OR EXCLUDE (EXACTSRCTITLE , “Seminars in Perinatology”) OR EXCLUDE (EXACTSRCTITLE , “Children”)) AND (EXCLUDE (EXACTKEYWORD , “Neonatal Respiratory Distress Syndrome”) OR EXCLUDE (EXACTKEYWORD , “Infant, Premature”) OR EXCLUDE (EXACTKEYWORD , “Pediatric Intensive Care Unit”) OR EXCLUDE (EXACTKEYWORD , “Prematurity”) OR EXCLUDE (EXACTKEYWORD , “Meta Analysis”) OR EXCLUDE (EXACTKEYWORD , “Observational Study”) OR EXCLUDE (EXACTKEYWORD , “Infant”) OR EXCLUDE (EXACTKEYWORD , “Infant, Newborn”) OR EXCLUDE (EXACTKEYWORD , “Retrospective Study”))
Cochrane Library	#1 (extubation):ti,ab,kw; #2 (postextubation failure):ti,ab,kw; #3 (respiratory failure postextubation):ti,ab,kw; #4 (extubation failure):ti,ab,kw; #5 (“noninvasive mechanical ventilation”):ti,ab,kw; #6 (“NIMV”):ti,ab,kw; #7 (high flow nasal cannula):ti,ab,kw; #8 (hfnc):ti,ab,kw; #9 (high flow):ti,ab,kw; #10 (“extubate”):ti,ab,kw; #11 (“extubating”):ti,ab,kw; Final combination: (#1 OR #12 OR #13) AND (#2 OR #3 OR #4) AND (#5 OR #6 OR #7 OR #8 OR #9 OR #10 OR #11) NOT (#13 OR #15)
Google Scholar	“extubation failure” AND (“high flow nasal cannula” OR “noninvasive ventilation”) AND “randomized controlled trial” AND (“ICU” OR “critical care”) -pediatric -neonatal -child -children -pregnancy -infant -pilot -protocol -review
Clinical Trials	“extubation failure” AND(“high flow nasal cannula” OR “noninvasive ventilation”)and(“randomized controlled trial” OR RCT OR “clinical trial”)
GrayNet International	“extubation failure” “high flow nasal cannula” OR “noninvasive ventilation” “clinical trial” -pediatric -neonatal -pregnancy
Open Gray	“extubation failure” “high flow nasal cannula” OR “noninvasive ventilation” “clinical trial” -pediatric -neonatal -pregnancy

This is an open access article under the CC BY license (https://creativecommons.org/licenses/by/4.0/).

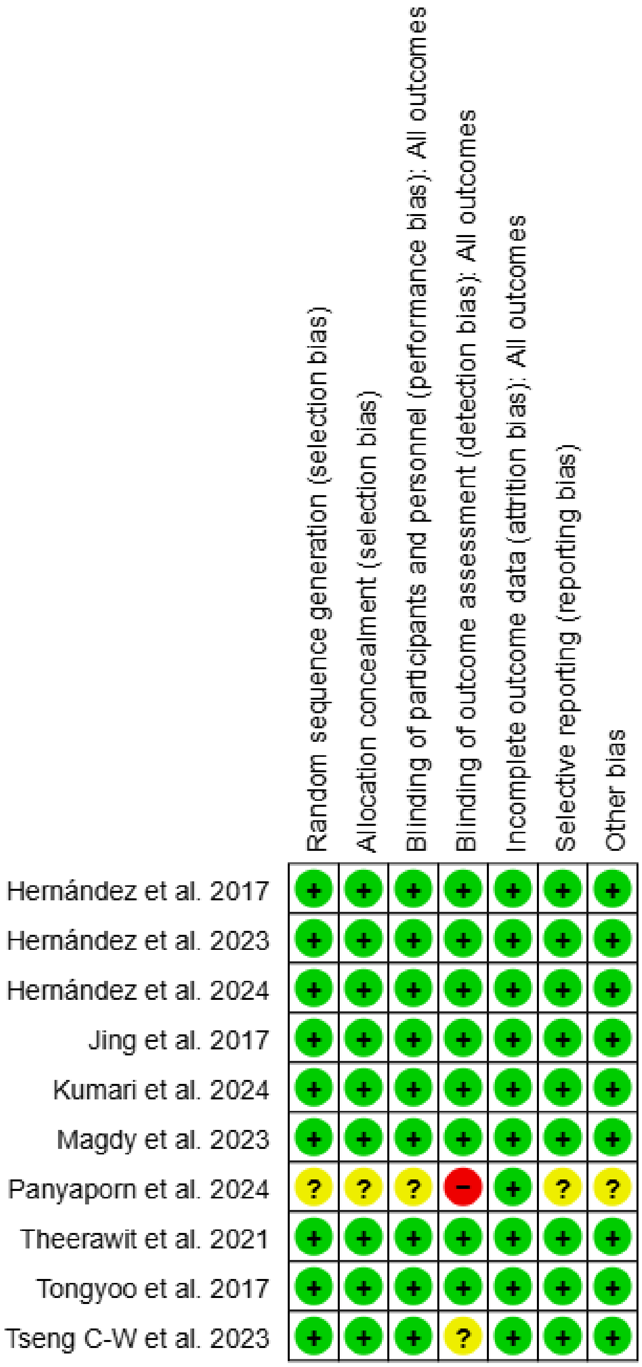

Figure 1S - Risk of bias assessment of the included randomized controlled trials using the Rob 2 tool.

Each domain represents a potential source of bias, and judgments are presented as "low risk", "some concerns", or "high risk" according to the Cochrane risk of bias 2.0 criteria.

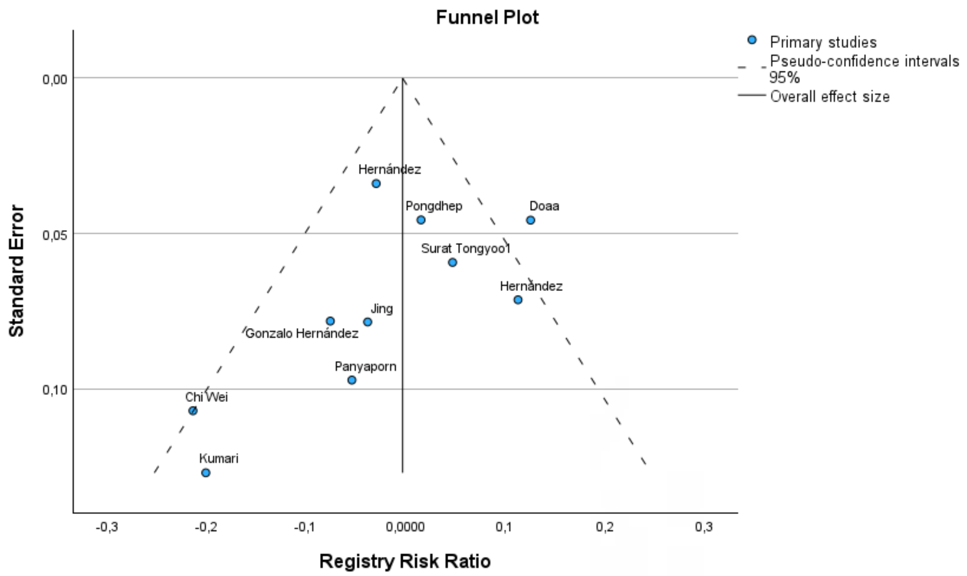

Figure 2SA - Funnel plot for the risk of reintubation comparing high-flow nasal cannula *versus* noninvasive ventilation.

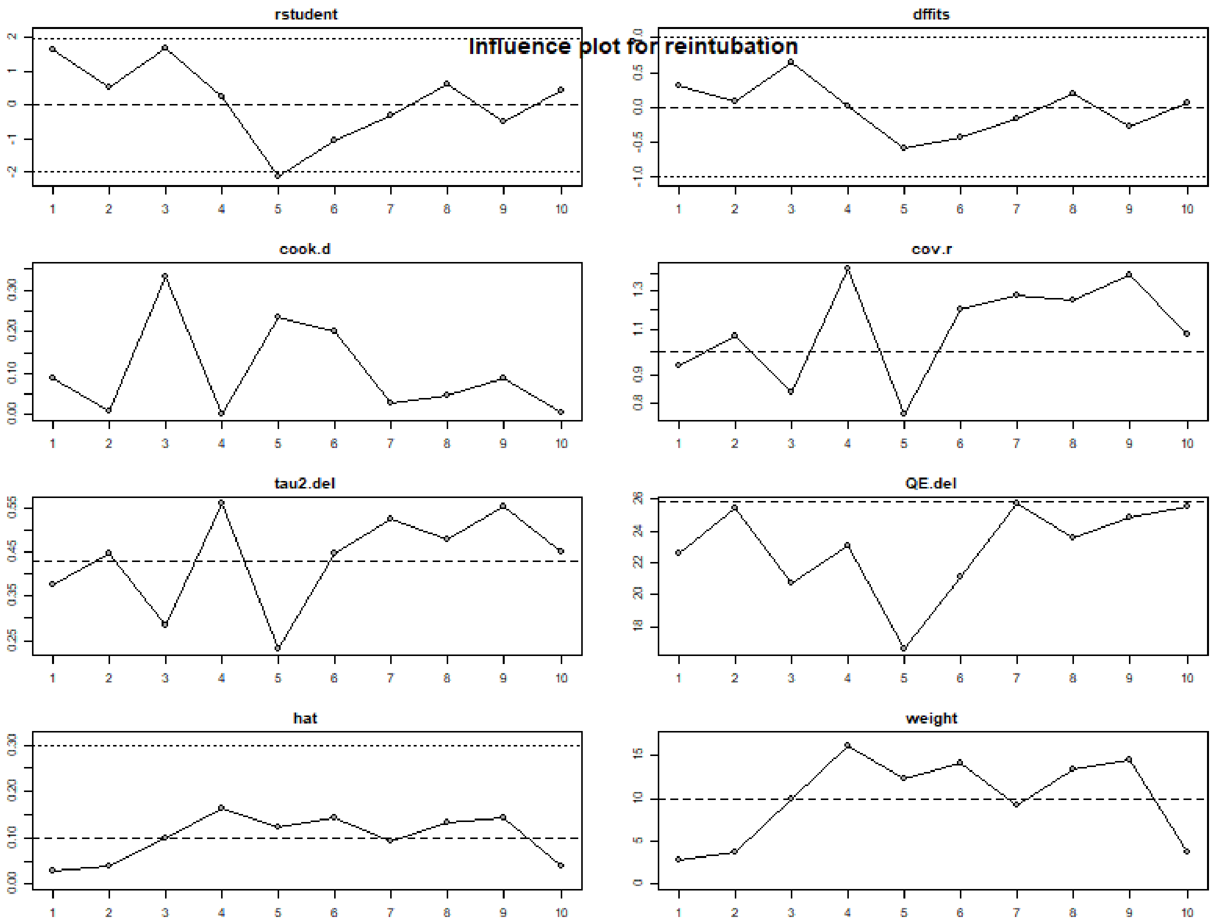

Figure 2SB - Influence plot illustrating the effect of individual studies on reintubation.

Each point represents an individual study included in the meta-analysis, numbered 1 to 10 (see Table 1 for study details).

The plot shows the influence of each study on the overall effect estimate; points farther from the center indicate a greater impact. Study 5[34] (Magdy et al.) was identified as a potential outlier, suggesting that heterogeneity may be partly due to differences in patient characteristics and study design.

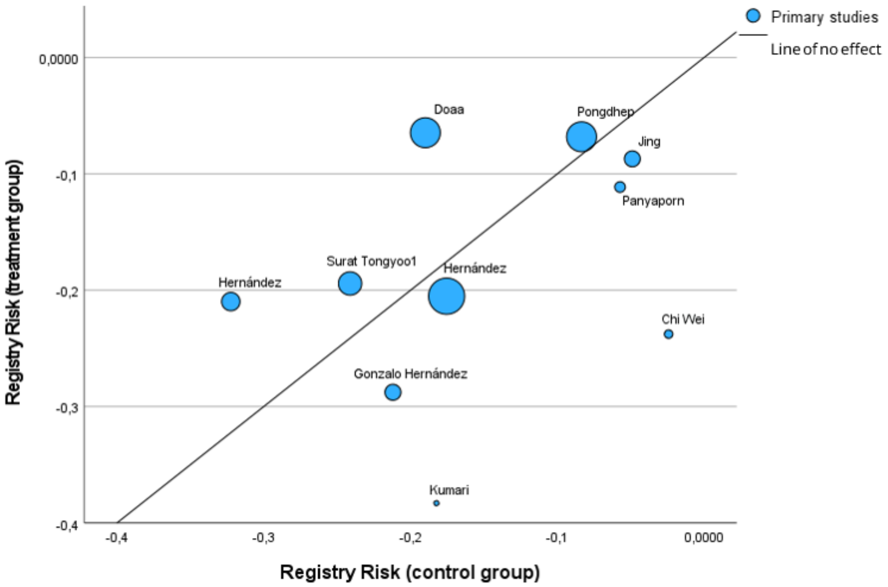

Figure 3S - L´Abbé plot of reintubation outcomes comparing high-flow nasal cannula and noninvasive ventilation.

Studies are plotted by reintubation rate in both arms. The diagonal line indicates equal event rates between groups. Points below the line favor high-flow nasal cannula, while points above favor noninvasive ventilation.

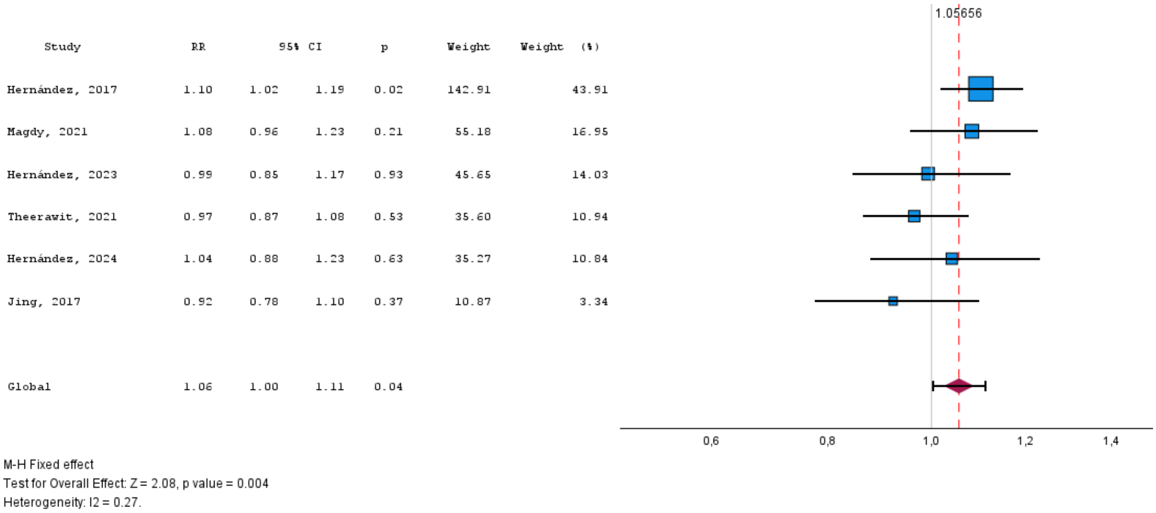

Study	RR	95% CI		p	Weight	Weight (%)
Hernández, 2017	1.10	1.02	1.19	0.02	142.91	43.91
Magdy, 2021	1.08	0.96	1.23	0.21	55.18	16.95
Hernández, 2023	0.99	0.85	1.17	0.93	45.65	14.03
Theerawit, 2021	0.97	0.87	1.08	0.53	35.60	10.94
Hernández, 2024	1.04	0.88	1.23	0.63	35.27	10.84
Jing, 2017	0.92	0.78	1.10	0.37	10.87	3.34
Global	1.06	1.00	1.11	0.04		

M-H Fixed effect
Test for Overall Effect: Z = 2.08, p value = 0.004
Heterogeneity: I2 = 0.27.

Figure 4S - Forest plot comparing the effect of high-flow nasal cannula *versus* noninvasive ventilation for preventing post-extubation respiratory failure.

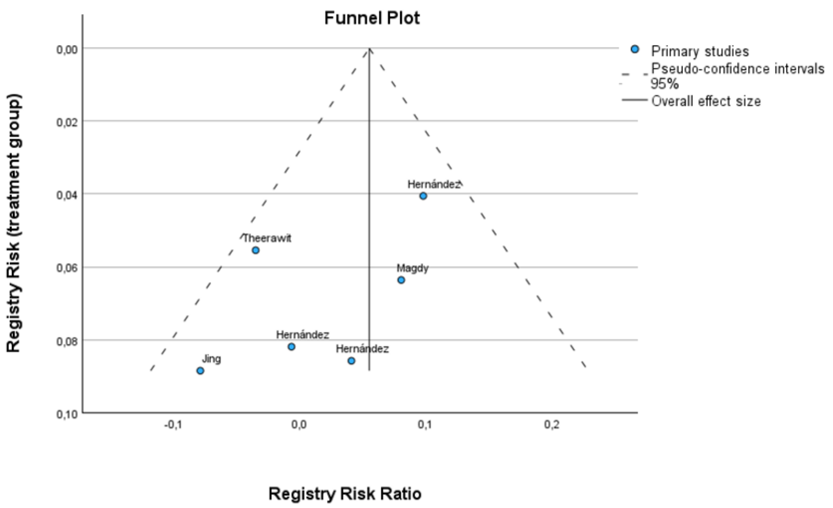

Figure 5SA - Funnel plot assessing publication bias for studies reporting post-extubation respiratory failure.

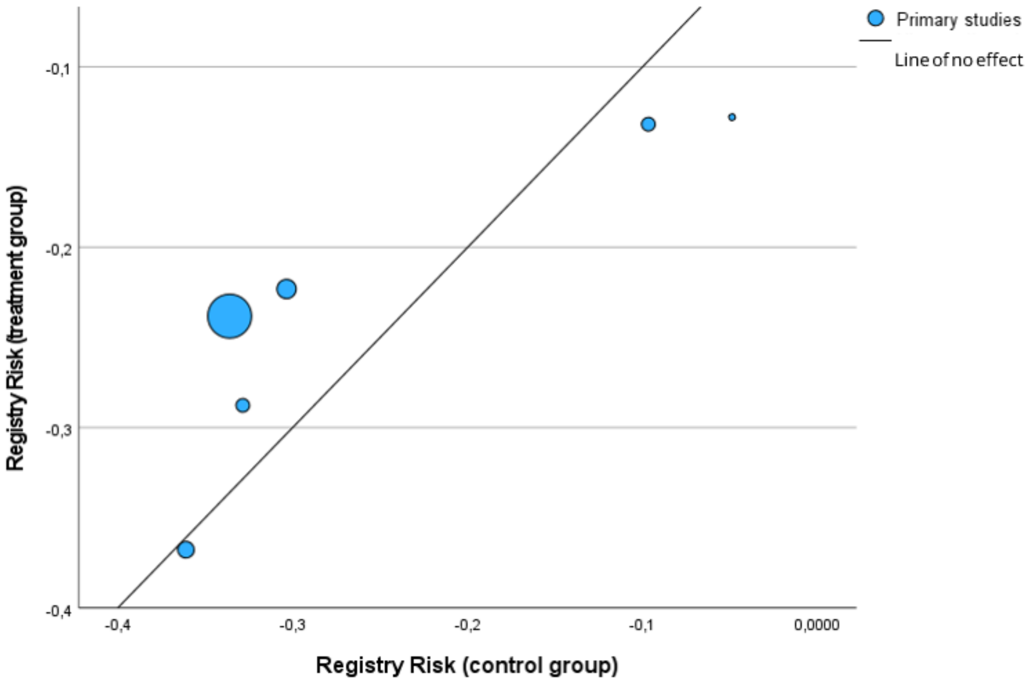

Figure 5SB - L´Abbé plot of post-extubation respiratory failure outcomes comparing high-flow nasal cannula and noninvasive ventilation.
The diagonal line indicates equal event rates between groups. Points below the line favor high-flow nasal cannula, while points above favor noninvasive ventilation.

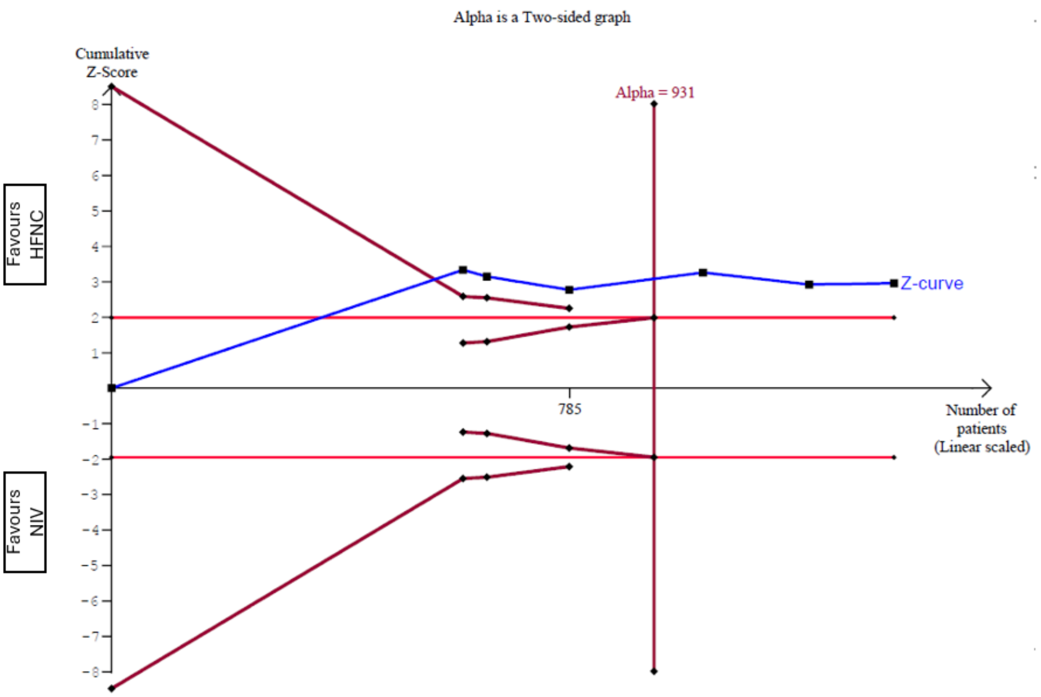

NIV - noninvasive ventilation; HFNC - high-flow nasal cannula.

Figure 6S - Trial sequential analysis of post-extubation respiratory failure.

The cumulative Z-curve (blue line) crossed the required information size (vertical red line at 931 patients), without reaching the trial sequential monitoring boundaries (red lines). A random-effects model was applied, assuming a type I error of 5%, 80% statistical power, and a 20% relative risk reduction. These results indicate that the required information size has been achieved, and further trials are unlikely to change the conclusion that a 20% relative risk reduction in post-extubation respiratory failure is not present.

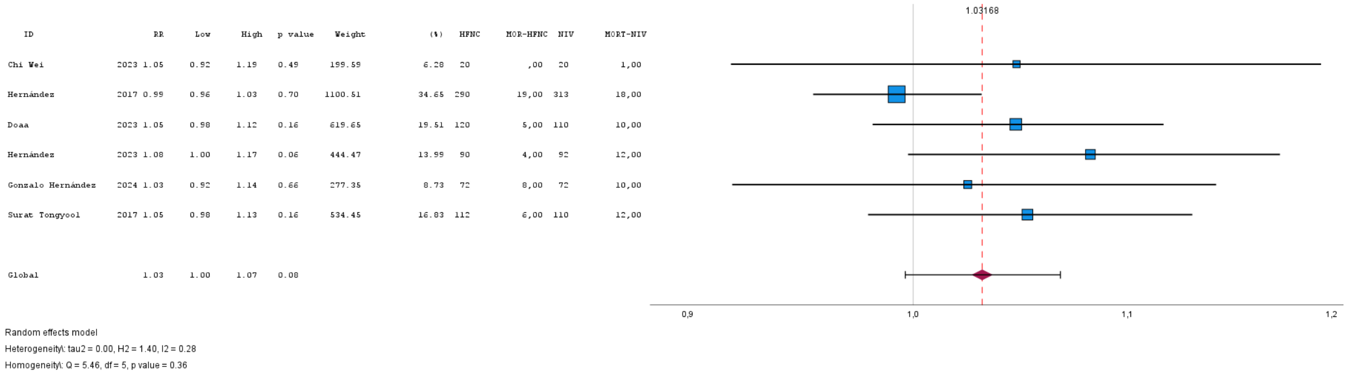

HFNC - high-flow nasal cannula; NIV - noninvasive ventilation.

Figure 7SA - Forest plot comparing the effect of high-flow nasal cannula *versus* noninvasive ventilation in intensive care unit mortality.

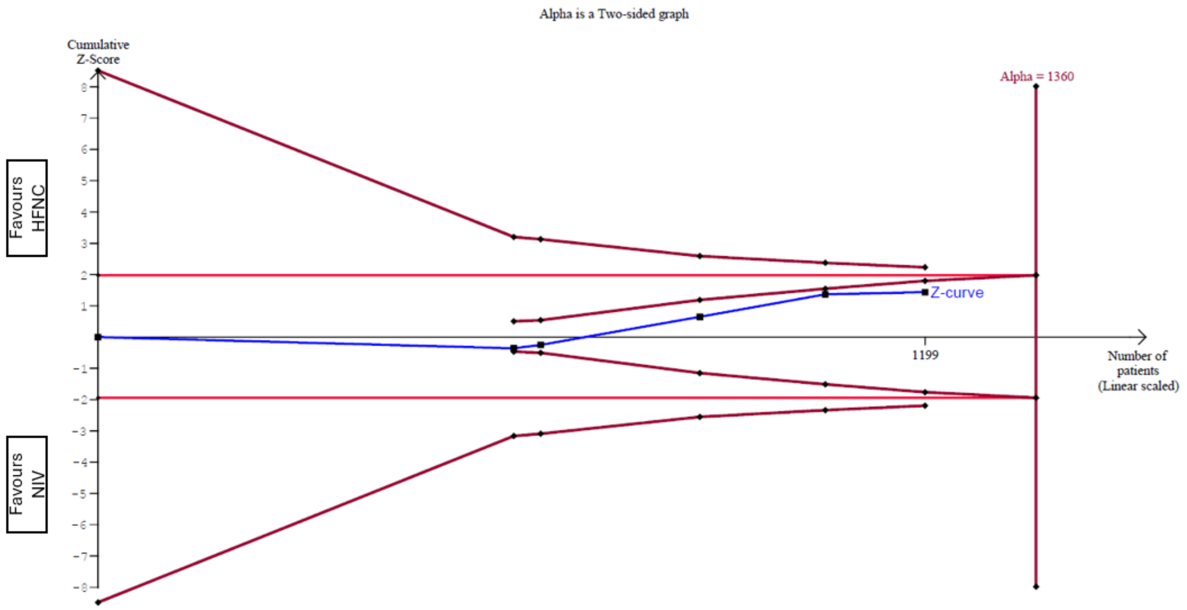

NIV - noninvasive ventilation; HFNC - high-flow nasal cannula.

Figure 7SB - Trial sequential analysis of intensive care unit mortality.

The cumulative Z-curve (blue line) did not cross the required information size (vertical red line at 1,360 patients) and did not reach the trial sequential monitoring boundaries (red lines). A random-effects model was applied, assuming a type I error of 5%, 80% statistical power, and a 20% relative risk reduction. These results indicate that despite nearly reaching the required information size, the available evidence is insufficient to confirm or reject a 20% relative risk reduction in intensive care unit mortality.

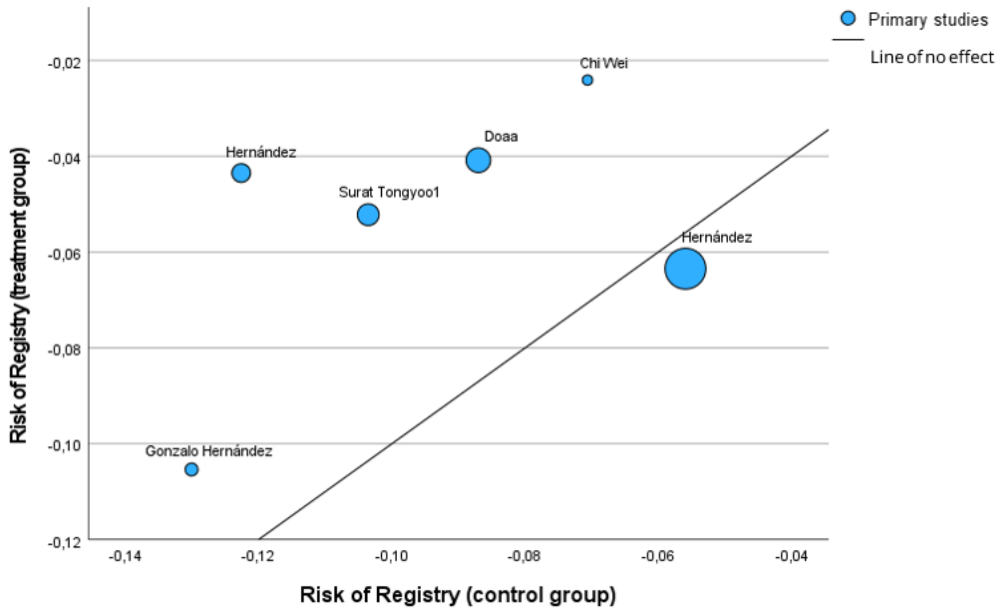

Figure 7SC - L´Abbé plot of intensive care unit mortality outcomes comparing high-flow nasal cannula and noninvasive ventilation.

The diagonal line indicates equal event rates between groups. Points above the line favor high-flow nasal cannula, while points below favor noninvasive ventilation.

Study	RR	CI 95% M-H fixed		p	Weight (%)	HFNC	MORT-HFNC	NIV	MORT-NIV
Tseng, 2023	1.09	0.87	1.36	0.44	2.02	20	2	20	4
Hernández, 2017	0.98	0.92	1.04	0.53	24.33	290	59	313	56
Magdy, 2023	1.06	0.99	1.13	0.11	22.40	120	5	110	11
Hernández, 2023	1.08	0.99	1.18	0.09	12.20	90	6	92	14
Theerawit, 2021	1.00	0.91	1.11	0.96	10.00	71	7	69	7
Hernández, 2024	1.03	0.92	1.14	0.66	8.43	72	8	72	10
Tongyoo, 2017	1.02	0.95	1.09	0.62	19.21	112	8	110	10
Jing, 2019	1.02	0.78	1.33	0.89	1.41	22	5	20	5
Global	1.02	0.99	1.06	0.13					

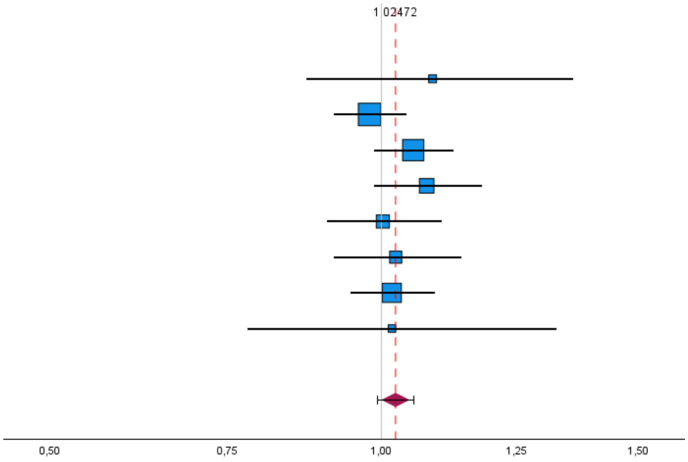

Heterogeneity: H2 = 0.64, I2 = 0.00
Homogeneity: Q = 4.50, df = 7, p = 0.72
Test for overall effect: Z = 1.51, p = 0.13

HFNC - high-flow nasal cannula; NIV - noninvasive ventilation.

Figure 8SA - Forest plot comparing the effect of high-flow nasal cannula *versus* noninvasive ventilation in hospital mortality.

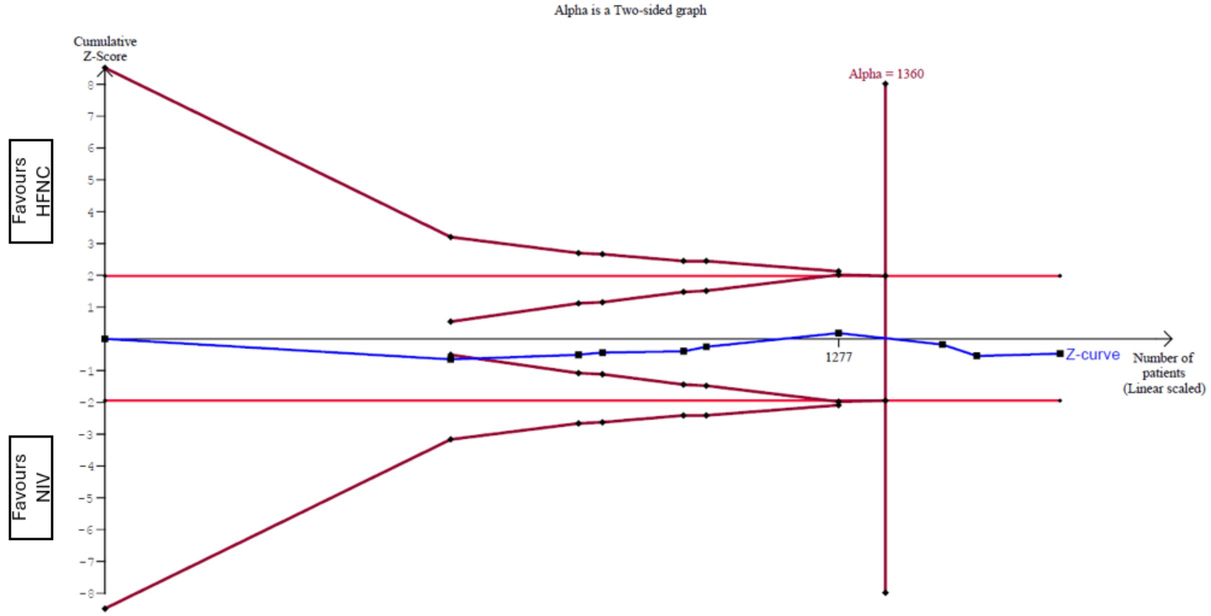

HFNC - high-flow nasal cannula; NIV - noninvasive ventilation.

Figure 8SB - Trial sequential analysis of hospital mortality.

The cumulative Z-curve (blue line) crossed the required information size (vertical red line at 1,360 patients), without reaching the trial sequential monitoring boundaries (red lines). A random-effects model was applied, assuming a type I error of 5%, 80% statistical power, and a 20% relative risk reduction. These results indicate that the required information size has been achieved, and further trials are unlikely to change the conclusion that a 20% relative risk reduction in hospital mortality.

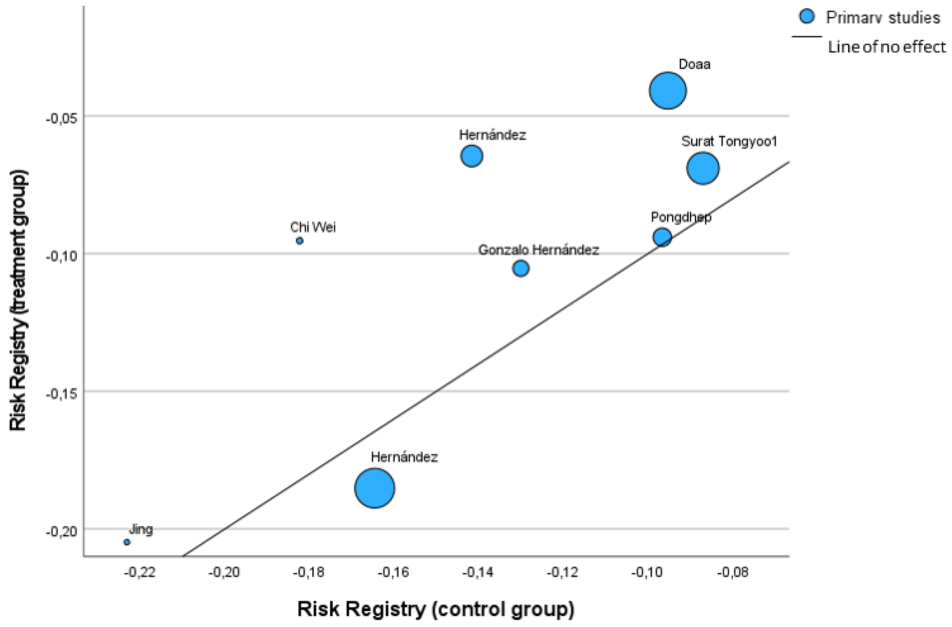

Figure 8SC - L´Abbé plot of hospital mortality outcomes comparing high-flow nasal cannula and noninvasive ventilation.
The diagonal line indicates equal event rates between groups. Points above the line favor high-flow nasal cannula, while points below favor noninvasive ventilation.

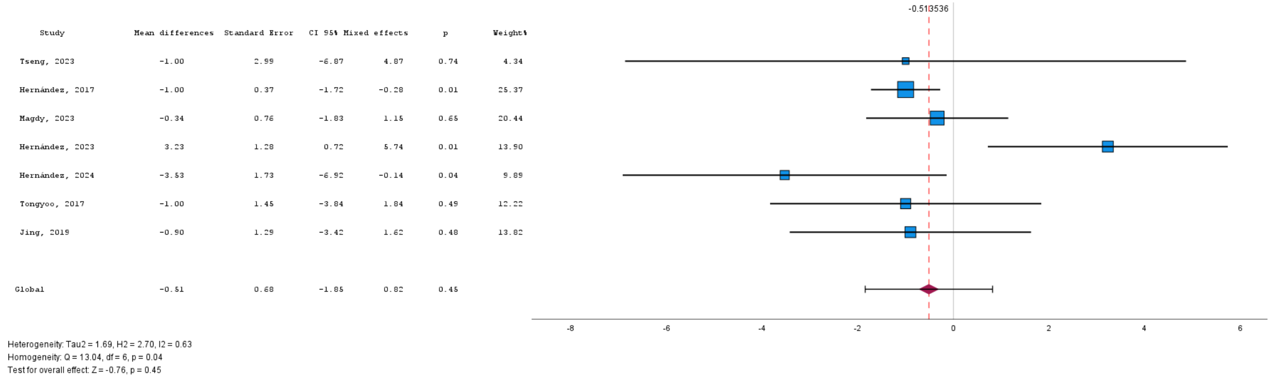

Figure 9SA - Forest plot comparing the effect of high-flow nasal cannula *versus* noninvasive ventilation on the length of stay in the intensive care unit.

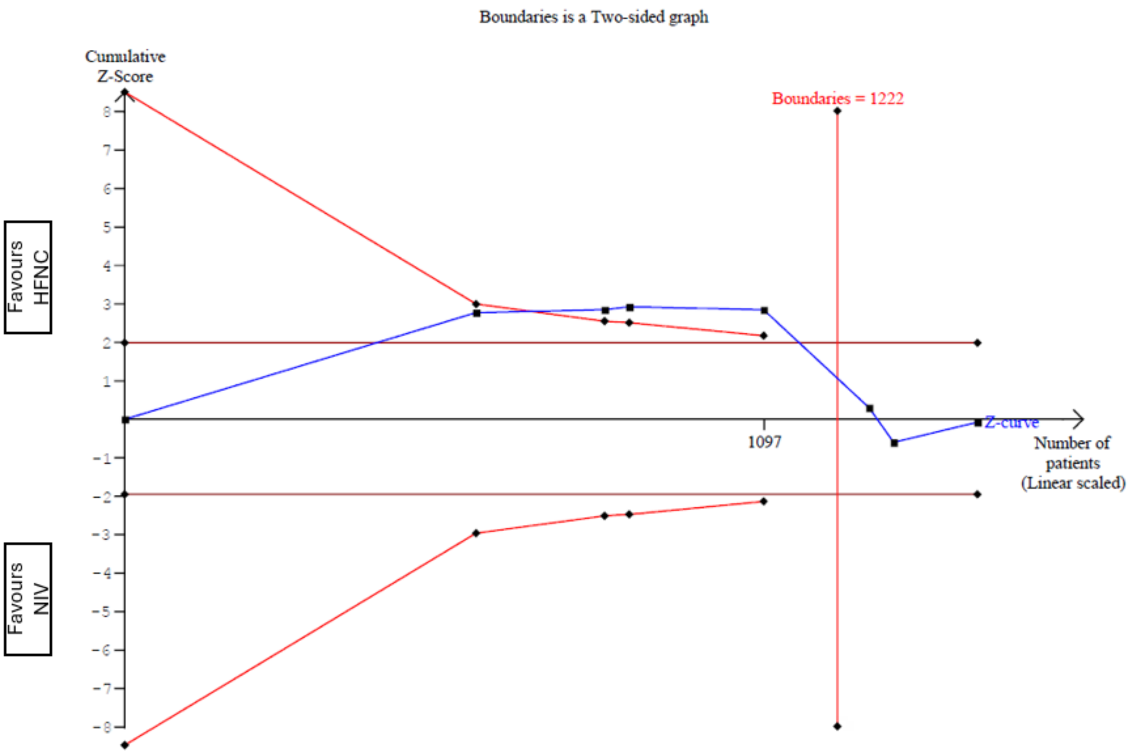

HFNC - high-flow nasal cannula; NIV - noninvasive ventilation.

Figure 9SB - Trial sequential analysis of length of stay at intensive care unit.

The cumulative Z-curve (blue line) crossed the required information size (vertical red line at 1,222 patients), without reaching the trial sequential monitoring boundaries (red lines). A random-effects model was applied, assuming a type I error of 5%, 80% statistical power, and a mean difference of 2 days. These results indicate that the required information size has been achieved, and further trials are unlikely to change the conclusion.

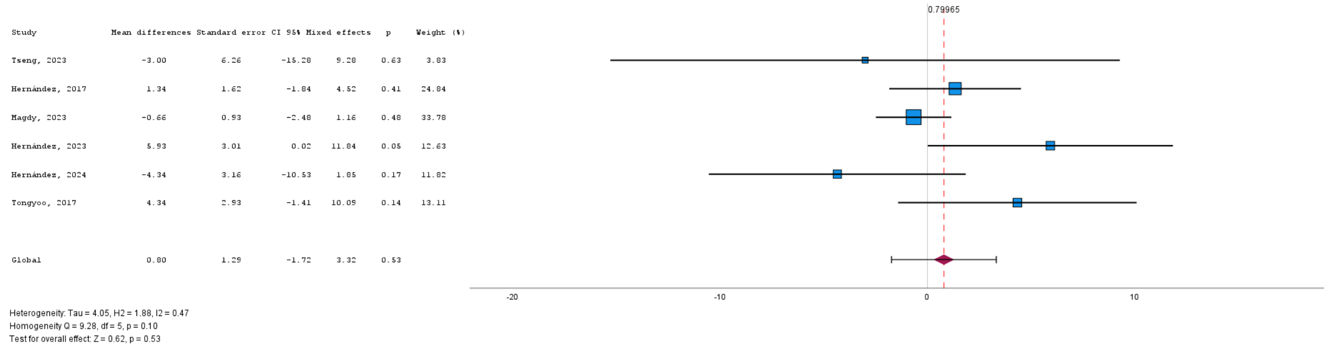

Figure 10SA - Forest plot comparing the effect of high-flow nasal cannula *versus* noninvasive ventilation on the length of stay in hospital.

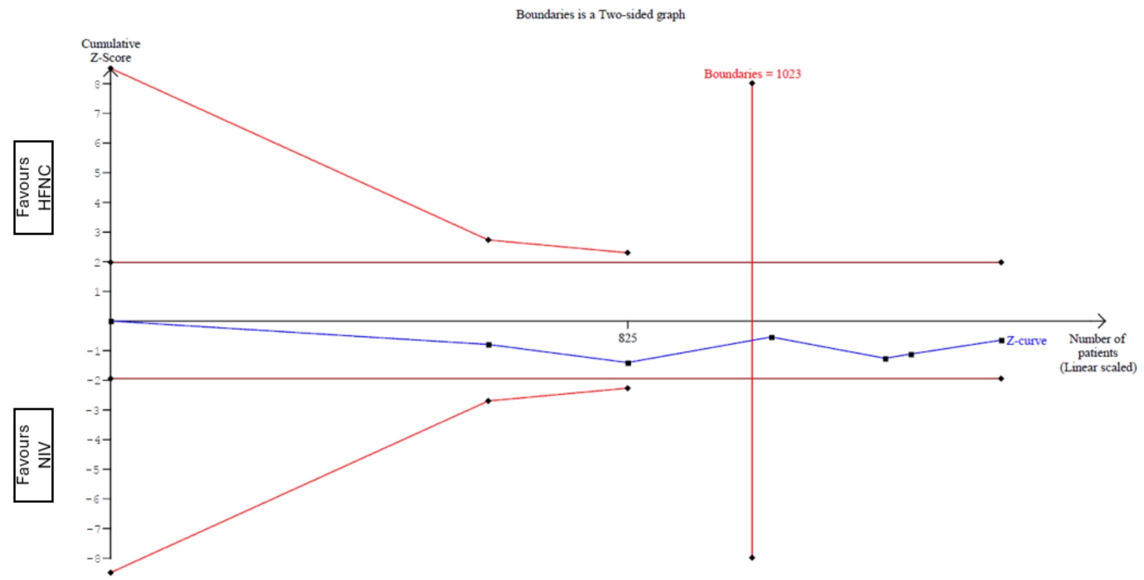

HFNC - high-flow nasal cannula; NIV - noninvasive ventilation.

Figure 10SB - Trial Sequential Analysis of length of stay at hospital.

The cumulative Z-curve (blue line) crossed the required information size (vertical red line at 1,023 patients), without reaching the trial sequential monitoring boundaries (red lines). A random-effects model was applied, assuming a type I error of 5%, 80% statistical power, and a mean difference of 5 days. These results indicate that the required information size has been achieved, and further trials are unlikely to change the conclusion.

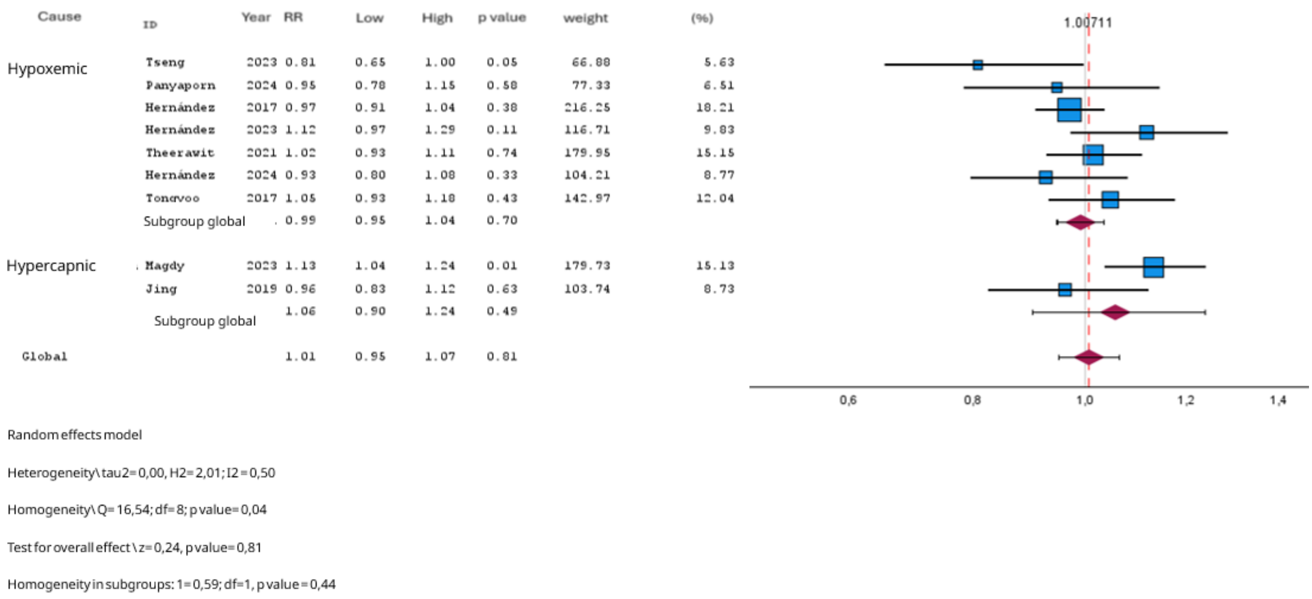

Figure 11S - Forest plot comparing the effect of high-flow nasal cannula *versus* noninvasive ventilation on reintubation stratified by cause of intubation.

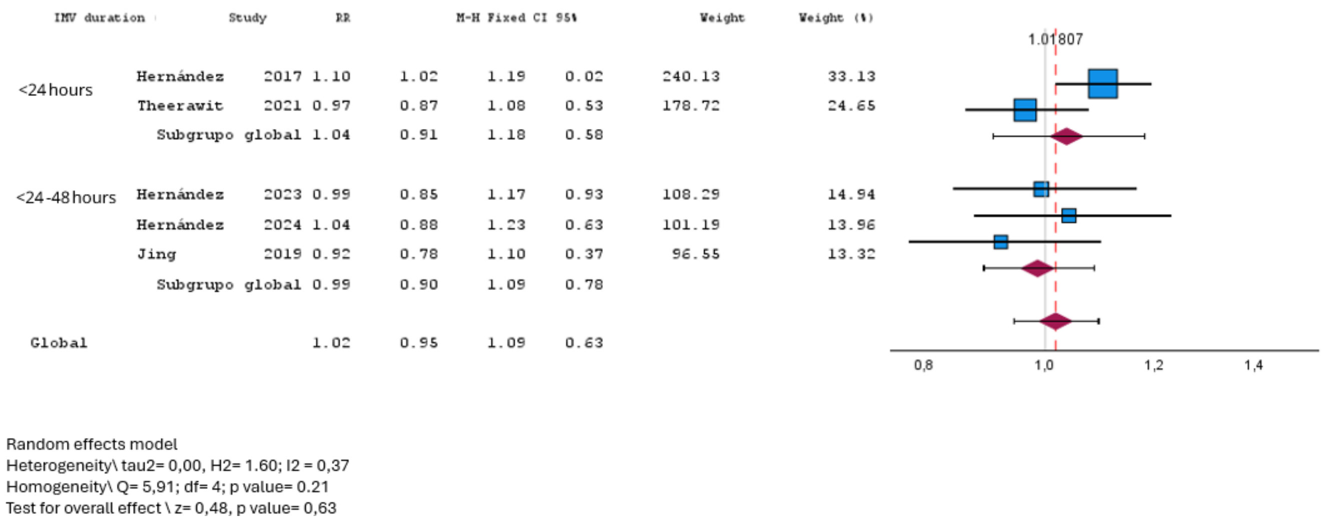

Figure 12S - Forest plot comparing the effect of high-flow nasal cannula *versus* noninvasive ventilation on post-extubation respiratory failure, stratified by the duration of treatment.

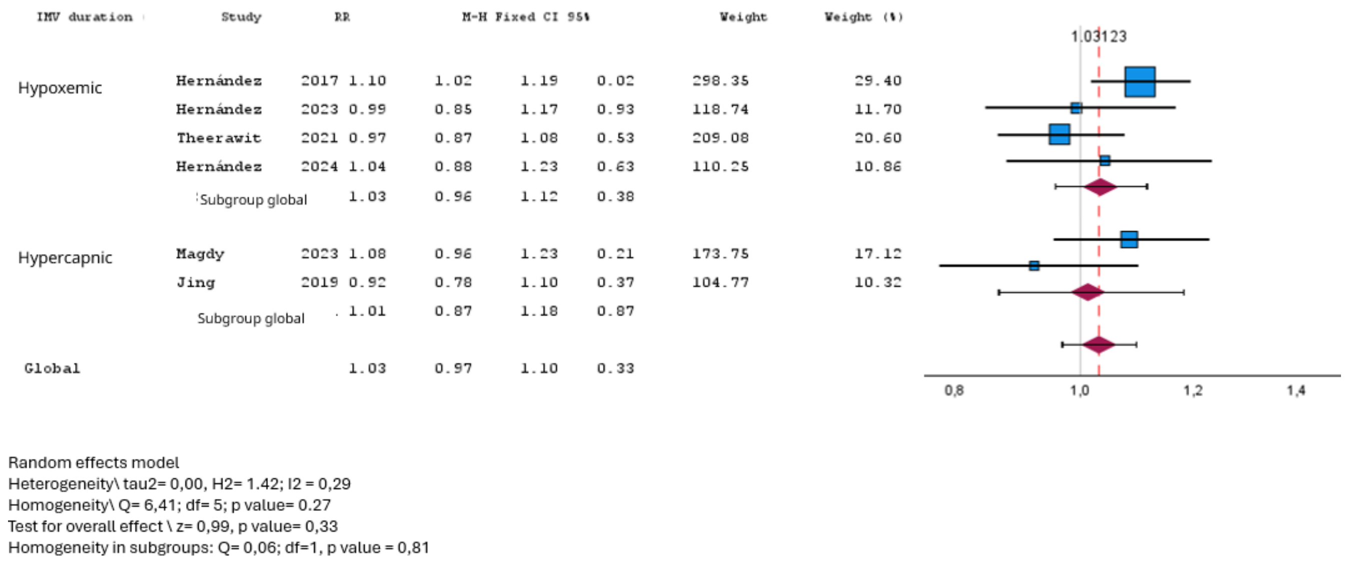

Figure 13S - Forest plot comparing the effect of high-flow nasal cannula *versus* noninvasive ventilation on post-extubation respiratory failure, stratified by cause of intubation.

IMV duration (days)	Study	RR	M-H Fixed CI 95%			Weight	Weight (%)
3-5	Hernández, 2017	1.10	1.02	1.19	0.02	142.91	43.91
	Hernández, 2023	1.04	0.88	1.23	0.63	35.27	10.84
	Jing, 2017	0.92	0.78	1.10	0.37	10.87	3.73
Subgroup global		1.08	1.01	1.16	0.03		
5-7	Magdy, 2023	1.08	0.96	1.23	0.21	55.18	16.95
	Hernández, 2024	0.99	0.85	1.17	0.93	45.65	14.03
	Theerawit, 2021	0.97	0.87	1.08	0.53	35.60	10.94
Subgroup global		1.02	0.94	1.11	0.58		
Global		1.06	1.00	1.11	0.04		

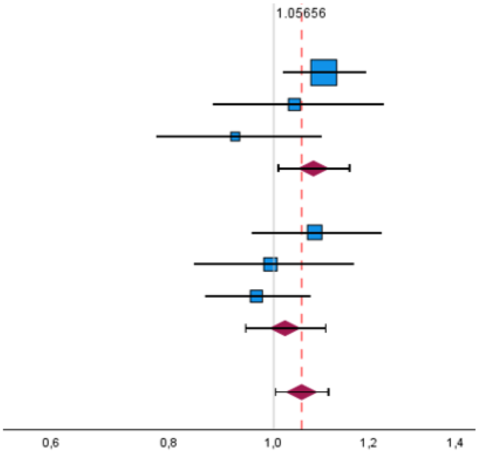

Figure 14S - Forest plot comparing the effect of high-flow nasal cannula *versus* noninvasive ventilation on post-extubation respiratory failure, stratified by the duration of invasive mechanical ventilation.

Table 2S - Results of meta-regression analysis evaluating the association between the number of risk factors and the risk of reintubation

Number of risk factors	Studies (k)	Estimate (propA)	SE	z-value	p-value	95%CI	I^2 (%)
1	9	-0.2220	0.5435	-0.4085	0.6829	-1.2874 - 0.8433	75.43
2 - 3	9	0.0042	0.0125	0.3326	0.7394	-0.0203 - 0.0286	71.27
≥ 4	9	0.1747	0.6765	0.2582	0.7963	-1.1513 - 1.5006	76.86

K - number of studies included in the meta-regression analysis; propA - proportion of patients with the covariate (moderator) in each study; SE - standard error; 95%CI - 95% confidence interval; I^2 - percentage of total variability due to heterogeneity rather than chance.

Table 3S - Results of subgroup and sensitivity analyses for the outcomes of reintubation, post-extubation respiratory failure, intensive care unit mortality and 28-day mortality

Subgroup	Studies	Participants	Risk ratio (95%CI)	p-value	Heterogeneity I^2% (τ^2)
Reintubation					
IMV (days)					
≤ 2	2	94	0.90 (0.77 - 1.04)	0.16	0 (0)
3 - 5	3	789	0.96 (0.91 - 1.02)	0.46	0 (0)
5 - 7	4	774	1.07 (1.01 - 1.14)	0.02	22 (0.001)
≥ 7	1	40	0.81 (0.65 - 1.00)	0.05	NA (NA*)
Intervention duration (hours)					
< 24	5	1,235	0.77 (0.46 - 1.29)	0.36	0 (0)
< 48	4	428	1.22 (059 - 2.50)	0.59	49.5 (0.007)
Causes of intubation					
Hypoxemic respiratory failure	7	1,365	0.99 (0.95 - 1.04)	0.7	6.1 (0)
Hypercapnic respiratory failure	2	272	1.06 (0.90 - 1.24)	0.49	70 (0.009)
Sensitivity analysis (excluding Panyaporn et al.)	9	1,637	1 (0.94 - 1.06)	0.98	58 (0)
Sensitivity analysis (excluding Magdy et al.)	9	1,437	0.98 (0.95 - 1.02)	0.44	0 (0)
Post-extubation respiratory failure					
IMV (days)					
3 - 5	3	828	0.93 (0.86 - 0.99)	0.03	32 (NA*)
5 - 7	3	514	0.98 (0.90 - 1.06)	0.58	0 (NA*)
Intervention duration (hours)					
< 24	2	743	1.04 (0.91 - 1.18)	0.53	58 (0.006)
< 48	3	368	0.99 (0.90 - 1.09)	0.78	0 (0)
Causes of intubation					
Hypoxemic respiratory failure	4	1,069	1.03 (0.97 - 1.13)	0.38	36 (0.002)
Hypercapnic respiratory failure	2	272	1.01 (0.87 - 1.18)	0.87	53 (0.007)
Sensitivity analysis (excluding Magdy et al.)	5	1,112	1.02 (0.95 - 1.12)	0.63	37 (0.002)
ICU mortality					
IMV (days)					
3 -5	2	747	1.01 (0.96 - 1.03)	0.83	0 (0)

Continue...

...continuation

5 - 7	3	634	1.06 (1.02 - 1.10)	0.01	0 (0)
≥ 7	1	40	1.06 (0.92 - 1.19)	0.49	NA (NA*)
Intervention duration (hours)					
< 24	3	865	1.1 (0.98 - 1.04)	0.63	15 (NA*)
< 48	2	326	1.06 (0.99 - 1.13)	0.07	0 (NA*)
Causes of intubation					
Hypoxemic respiratory failure	5	1,191	1.03 (0.99 - 1.07)	0.17	33 (0.001)
Hypercapnic respiratory failure	1	230	1.05 (0.98 - 1.12)	0.16	NA (NA*)
Sensitivity analysis (excluding Magdy et al.)	5	1,191	1.03 (0.99 - 1.07)	0.17	33 (0.001)
28-days mortality					
IMV (days)					
3 - 5	3	799	0.99 (0.94 - 1.05)	0.78	0 (0)
5 - 7	4	781	1.04 (1 - 1.08)	0.05	0 (0)
≥ 7	1		1.09 (0.87 - 1.36)	0.44	NA (NA*)
Intervention duration (hours)					
< 24	4	1,005	1 (0.96 - 1.04)	0.97	0 (0)
< 48	3	368	1.06 (0.99 - 1.13)	0.39	0 (0)
Causes of intubation					
Hypoxemic respiratory failure	6	1,331	1.02 (0.98 - 1.05)	0.40	0 (0)
Hypercapnic respiratory failure	2	272	1.06 (0-99 - 1.12)	0.11	0 (0)
Sensitivity analysis (excluding Magdy et al.)	7	1373	1.02 (0.98 - 1.06)	0.39	0 (0)

95%CI - 95% confidence interval; IMV - invasive mechanical ventilation; ICU – intensive care unit; NA - no available; NA* - not applicable (fixed-effects model).

Table 4S - Results of subgroup and sensitivity analyses for the outcomes of lengths of stay in the intensive care unit and hospital

Subgroup	Studies	Participants	Mean differences (95%CI)	p-value	Heterogeneity (I2)
Length of stay in intensive care unit					
IMV (days)					
3 - 5	3	789	-1.10 (-0.70 - [-1.41])	0.001	0
5 - 7	3	635	0.59 (-0.86 - 3.04)	0.64	72
≥ 7	1	40	-0.51 (-1.85 - 0.82)	0.45	NA
Intervention duration (hours)					
< 24	3	865	-1 (-0.7 - [-0.30]	0.001	0
< 48	3	368	-0.28 (-4.10 - 3.54)	0.89	82
Causes of intubation					
Hypoxemic respiratory failure	5	1,191	-0.52 (-2.75 - 1.71)	0.65	74
Hypercapnic respiratory failure	2	272	-0.48 (-1.77 - 0.80)	0.46	0
Sensitivity analysis (excluding Magdy et al.)	6	1 233	-0.57 (-2.34 - 1.19)	0.53	68
Length of stay in hospital					
IMV (days)					
3 - 5	2	747	-0.85 (-6.27 - 4.57)	0.76	61
5 - 7	3	636	2-43 (-1.95 - 6.80)	0.28	66
≥ 7	1	40	-3 (-15.28 - 9.28)	0.63	NA
Intervention duration (hours)					
< 24	3	865	1.80 (-0.92 - 4.51)	0.19	0
< 48	2	326	0.84 (-9.23 - 10.9)	0.87	82
Causes of intubation					
Hypoxemic respiratory failure	5	1 191	1.50 (-2.01 - 5.01)	0.40	45
Hypercapnic respiratory failure	1	230	-0.66 (-2.48 - 1.16)	0.48	NA
Sensitivity analysis (excluding Magdy et al.)	5	1 191	1.50 (-2.01 - 5.01)	0.40	45

95%CI - 95% confidence interval; IMV - invasive mechanical ventilation; NA - no available.
